# The Impact of a Laboratory Error on a Patient’s Well Being: A Case Report on Overdiagnosis and Its Consequent Iatrogenesis

**DOI:** 10.7759/cureus.84446

**Published:** 2025-05-20

**Authors:** Luís Laranjeira Correia, Joana Ferreira Vieira, Catarina Oliveira Neves, Maria de Freitas Domingues

**Affiliations:** 1 Family Medicine, Unidade de Saúde Familiar São Pedro da Cova, Unidade Local de Saúde Santo António, Porto, PRT; 2 Family Medicine, Unidade de Saúde Familiar São Pedro da Cova,Unidade Local de Saúde Santo António, Porto, PRT

**Keywords:** complementary diagnostic tests, error, overdiagnosis, overtreatment, quaternary prevention

## Abstract

Overdiagnosis, resulting from excessive use of diagnostic tests, can lead to unnecessary treatments and negatively impact patients' quality of life. This case highlights the importance of prescribing tests based on the best scientific evidence to avoid emotional and financial harm to patients while promoting quaternary prevention. A 77-year-old woman, undergoing surveillance for a thyroid nodule, mistakenly underwent a urinary cytology test, which revealed a suspicious finding. Due to this incidental result, the Family Physician referred her to a Urology consultation to rule out urothelial cancer. The subsequent investigations included renal and pelvic CT scans, urethrocystoscopy, and repeated urinary cytology, all of which yielded negative results for malignancy. Throughout this process, the patient experienced anxiety, sadness, and loss of appetite, which also affected her family life.

The test requested by the Family Physician was a non-cervicovaginal exfoliative cytology, which can encompass various types of cytology. However, due to a laboratory error, urinary cytology was performed instead of a fine-needle aspiration cytology (FNAC) of the thyroid. This mistake led to an unfounded suspicion of cancer, causing significant anxiety for the patient and her family. The cascade of invasive tests and the use of limited healthcare resources underscore the dangers of overdiagnosis and the challenge of maintaining healthcare system sustainability. This case illustrates the importance of quaternary prevention, encouraging a cautious approach to ordering diagnostic and therapeutic tests, as well as involving patients in informed decision-making regarding their health. The role of the Family Physician is crucial in understanding patients' concerns and needs, promoting a holistic, patient-centered approach to healthcare delivery.

## Introduction

Diagnostic tests are valuable tools for determining the correct diagnosis based on a patient’s clinical history and symptoms, facilitating medical decision-making. However, overdiagnosis refers to the detection of an alteration or disease that would not have caused any complaints or harm in the future. Apart from the waste of healthcare resources, overdiagnosis can harm patients by leading to overtreatment with associated risks, anxiety, depressive states related to the diagnosis, labeling, or financial burden. This case report illustrates the impact of diagnostic suspicion on the quality of life of an asymptomatic patient who underwent an unintended clinical test. It aims to emphasize the importance of prescribing diagnostic tests based on the best available scientific evidence, ensuring not only secondary prevention but also quaternary prevention [1-4].

## Case presentation

The patient is a 77-year-old retired cleaning worker, autonomous in daily living activities and cognitively intact. She was classified as having stage VIII in Duvall’s family life cycle, with a functional family Apgar score and Graffar class 3. Her medical history included dyslipidemia, type 2 diabetes mellitus, hypertension, ischemic stroke in 2009, and multinodular euthyroid goiter with a dominant 16 mm nodule on the right lobe. Family history included a son with Graves' disease, a daughter diagnosed with breast cancer at 55 years old, and a maternal aunt with a diagnosis of colorectal cancer at 40 years old. Her medication regimen included acetylsalicylic acid 100 mg daily, atorvastatin 20 mg daily, metformin 500 mg twice daily, and perindopril/indapamide 10 mg/2.5 mg daily. She has no smoking or drug use habits.

Due to the stability of her thyroid condition, she was discharged from the General Surgery outpatient clinic, with a recommendation for follow-up by the Family Physician, including annual ultrasound evaluations and fine-needle aspiration cytology (FNAC) if there was dimensional growth of the nodule. During a follow-up visit, the patient was asymptomatic, and a thyroid ultrasound was requested as per protocol. The report indicated a 2 mm increase in the dominant nodule, leading to an FNAC request via a non-cervicovaginal exfoliative cytology test, specifying that the sample was for a thyroid nodule biopsy.

**Figure 1:**
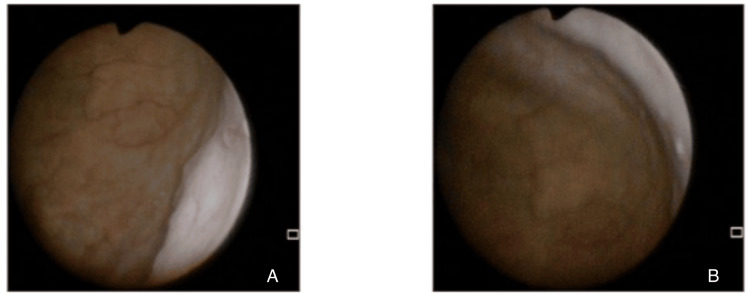
Endoscopic views obtained during urethrocystoscopy A: Lateral bladder wall with a uniformly smooth urothelium, a faint arborising vascular network, and no focal mucosal irregularities or suspicious lesions; B: Trigone and bladder-neck region with intact urothelium, adequate luminal distension, and no macroscopic evidence of inflammation, hyperplasia, or neoplastic growth

The patient later returned with the results of a mistakenly performed urinary cytology, which identified atypical urothelial cells (Paris classification). Despite the absence of urinary symptoms, due to this incidental finding, the Family Physician ordered a renal and pelvic CT scan and referred her to a Urology consultation to rule out urothelial malignancy. The imaging study showed no abnormalities in the urinary system. In the outpatient Urology consultation, the patient underwent urethrocystoscopy, which showed no alterations (Figure 1), and a repeat urinary cytology, which was negative for high-grade urothelial carcinoma (Paris classification). Consequently, she was discharged from the hospital consultation.

At a subsequent follow-up visit, the patient reported experiencing increased anxiety, sadness, and health-related concerns during the three-month period in which the suspicion of urothelial pathology loomed over her. She also described a decrease in appetite. Additionally, she mentioned that her family was significantly worried, especially her husband and children. However, after receiving the positive outcome, she noticed a gradual improvement in her emotional state and appetite. A physical examination revealed a weight loss of 4 kg since the initial evaluation.

## Discussion

The test requested by the Family Physician was a non-cervicovaginal exfoliative cytology. This type of test can refer to FNAC, exfoliative cytology, cytology of secretions, cytology of respiratory tract washes and brushings, or cytology of fluids (urine, ascitic fluid, or pleural fluid). Therefore, additional information is required to specify the intended test.

In this case, despite the correct specification in the diagnostic request, a laboratory error led to the performance of urinary cytology instead of FNAC. After undergoing an unintended diagnostic test, the patient was left with a suspicion of cancer despite having no personal or family history supporting such a diagnosis. The suspicion and subsequent excessive testing caused the patient and her family significant anxiety and distress. She underwent invasive tests with potential iatrogenic risks and misspent valuable healthcare resources in a National Health Service facing sustainability challenges [1].

## Conclusions

This case illustrates the impact of overdiagnosis, particularly in asymptomatic individuals, highlighting the importance of investing in quaternary prevention. To prevent excessive medical intervention and its consequent iatrogenesis, healthcare professionals should prioritize the judicious and economically rational prescription of diagnostic and therapeutic tests. Additionally, clear communication between the prescribing physician and the laboratory is essential, along with patient empowerment in seeking appropriate healthcare and understanding the benefits and drawbacks of diagnostic and therapeutic procedures. Furthermore, the role of the Family Physician is crucial in understanding patients' thoughts, expectations, emotions, and fears concerning diagnostic and therapeutic procedures. Physicians must be attentive to how patients perceive their health status and the impact it may have on their social and family lives. 
